# Non-invasive venous waveform analysis (NIVA) for volume assessment in patients undergoing hemodialysis: an observational study

**DOI:** 10.1186/s12882-020-01845-2

**Published:** 2020-05-24

**Authors:** Bret D. Alvis, Monica Polcz, Merrick Miles, Donald Wright, Mohammad Shwetar, Phil Leisy, Rachel Forbes, Rachel Fissell, Jon Whitfield, Susan Eagle, Colleen Brophy, Kyle Hocking

**Affiliations:** 1 Department of Anesthesiology, Division of Critical Care, Vanderbilt University Medical Center, 422 MAB, 1211 21st Ave South, Nashville, TN 37212 USA; 2 Vanderbilt University Medical Center, S111 Medical Center North, 21st Ave South, Medical Art Building 422, Nashville, TN 37212 USA; 3 Vanderbilt University School of Medicine, 1161 21st Ave S # D3300, Nashville, TN 37232 USA; 4 Department of Surgery, Division of Kidney and Pancreas Transplantation, Vanderbilt University Medical Center, 1301 Medical Center Drive, Nashville, TN 37232 USA; 5 Department of Medicine, Division of Nephrology and Hypertension, Vanderbilt University Medical Center, 1161 21st Ave South, MCN S-3223, Nashville, TN 37232 USA; 6 Volumetrix, LLC, 2126 21st Ave South, Nashville, TN 37212 USA

**Keywords:** Dialysis, Venous waveform analysis, Monitoring

## Abstract

**Background:**

Accurate assessment of volume status to direct dialysis remains a clinical challenge. Despite current attempts at volume-directed dialysis, inadequate dialysis and intradialytic hypotension (IDH) are common occurrences. Peripheral venous waveform analysis has recently been developed as a method to accurately determine intravascular volume status through algorithmic quantification of changes in the waveform that occur at different volume states. A noninvasive method to capture peripheral venous signals is described (Non-Invasive Venous waveform Analysis, NIVA). The objective of this proof-of-concept study was to characterize changes in NIVA signal with dialysis. We hypothesized that there would be a change in signal after dialysis and that the rate of intradialytic change in signal would be predictive of IDH.

**Methods:**

Fifty subjects undergoing inpatient hemodialysis were enrolled. A 10-mm piezoelectric sensor was secured to the middle volar aspect of the wrist on the extremity opposite to the access site. Signals were obtained fifteen minutes before, throughout, and up to fifteen minutes after hemodialysis. Waveforms were analyzed after a fast Fourier transformation and identification of the frequencies corresponding to the cardiac rate, with a NIVA value generated based on the weighted powers of these frequencies.

**Results:**

Adequate quality (signal to noise ratio > 20) signals pre- and post- dialysis were obtained in 38 patients (76%). NIVA values were significantly lower at the end of dialysis compared to pre-dialysis levels (1.203 vs 0.868, *p* < 0.05, *n* = 38). Only 16 patients had adequate signals for analysis throughout dialysis, but in this small cohort the rate of change in NIVA value was predictive of IDH with a sensitivity of 80% and specificity of 100%.

**Conclusions:**

This observational, proof-of-concept study using a NIVA prototype device suggests that NIVA represents a novel and non-invasive technique that with further development and improvements in signal quality may provide static and continuous measures of volume status to assist with volume directed dialysis and prevent intradialytic hypotension.

## Background

End-stage renal disease (ESRD) continues to represent a significant disease burden to patients and the health-care system. Approximately one-third of patients with ESRD will proceed to kidney transplantation while the remainder will require artificial renal-replacement therapy, most often accomplished via hemodialysis (HD). In 2016, 87.3% of incident individuals with ESRD began renal replacement therapy with HD, and 63.1% of all prevalent ESRD patients were receiving HD [1].

Comorbid cardiovascular conditions in the ESRD patient population are high, and this increases susceptibility to hemodynamic instability during HD, otherwise referred to as intradialytic hypotension (IDH). While there is no universal criteria for IDH, IDH is defined by the Kidney Disease Outcomes Quality Initiative (KDOQI) criteria as a decrease in systolic blood pressure by > 20 mmHg or a decrease in mean arterial blood pressure by > 10 mmHg associated with symptoms that include abdominal discomfort, fatigue, nausea, vomiting, muscle cramps, restlessness, dizziness, fainting, and anxiety [2]. IDH occurs because rapid fluid removal during HD depletes intravascular volume at a rate that exceeds that of secondary compartmental fluid shifts between the extravascular and intravascular compartments [3]. This poses a major challenge in that IDH frequently results in premature termination of hemodialysis despite an overall state of volume overload due to inadequate urine production. Finding the right pairing of optimal volume removal with appropriate volume removal rate is paramount to maintaining hemodynamic stability during treatments.

Assessment of volume status to direct dialysis is most commonly performed using objective clinical parameters such as vital signs or comparison of actual versus dry weight, as well as subjective measures including peripheral edema, jugular venous distention, lung auscultation and shortness of breath [3]. However, despite current attempts at volume-directed dialysis, IDH still occurs in up to 30% of HD sessions [4]. Consequences of IDH include premature termination of HD, end-organ hypoperfusion of the heart, gut, and brain and an overall increase in long-term mortality compared to patients without IDH [3, 5–8]. IDH remains the most common severe complication of HD, highlighting the difficulties in assessing intravascular volume status based on clinical parameters and demonstrating a clear need for more accurate volume assessment in this patient population.

Low amplitude venous waveforms have historically been largely ignored until appropriate sensing and amplifying technologies became available [9, 10]. Venous waveforms have recently been used to determine intravascular volume status [11–17]. The peripheral venous system is a highly compliant, low pressure system, and therefore serves as a volume reservoir storing 60–70% of the circulating blood volume [10, 18, 19]. Thus, changes in circulating blood volume are reflected first in the peripheral venous system. Systemic venous compliance is higher than systemic arterial compliance (146 vs 1.7 ml/mmHg) [20, 21], thus overall pressure is low and changes minimally with changes in volume, precluding absolute pressure measurements as an accurate measure of volume. However, analysis of the peripheral venous waveform in the frequency domain demonstrates characteristic changes in the amplitudes of the cardiac frequencies (corresponding to the pulse rate and its harmonics) with volume. The fundamental frequency of the venous signal (f_0_) corresponds to the pulse rate, and additional higher harmonics (f_1–2_) that are multiples of f_0_, are produced by interaction of the waves with the vein wall and surrounding tissues. Changes in the amplitudes of the cardiac frequencies relative to one another occur with changes in intravascular volume [22].

**Fig. 1:**
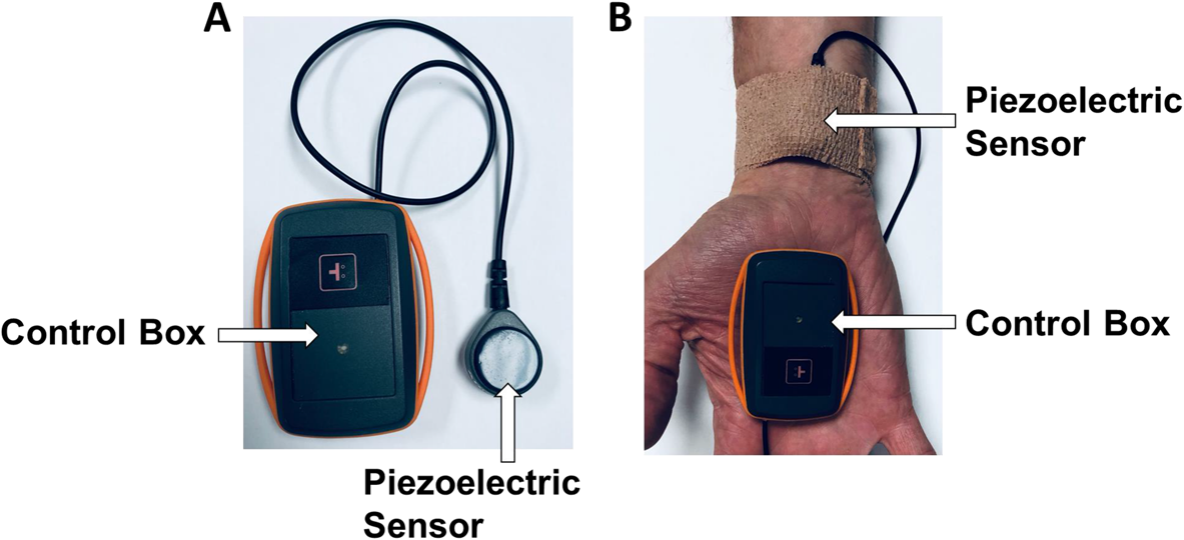
NIVA prototype. **(a)** The NIVA device consists of a piezo electric crystal sensor and housing control box that is **(b)** applied to the surface of the skin over the venous plexus at the volar aspect of the wrist. The current from the crystal is transferred via wire to the control box where the signal is amplified and converted to a digital signal and transferred via USB port to a computer for analysis

Initially, peripheral intravenous waveform analysis (PIVA) was used to obtain venous waveforms from an indwelling peripheral intravenous catheter [12, 14]. In patients undergoing hemodialysis, there is a linear correlation between PIVA signal and ultrafiltrate (ml/kg) removed (R^2^ = 0.77, *n* = 45 patients, *p* < 0.01) [11]. More recently, signals originating from the peripheral venous waveform have been obtained non-invasively using a piezo-electric sensor placed on the skin over the venous plexus on the volar aspect of the wrist (Non-Invasive Venous waveform Analysis, NIVA, Fig. 1). When placed directly over these superficial veins, the sensor is able to detect small deflections in the skin overlying the vein that occur with the cardiac cycle. This non-invasive technique is feasible because the algorithmic computation of a “NIVA value” utilizes the *proportional* amplitude of each cardiac frequency relative to the overall signal amplitude. Additionally, NIVA is more appropriate for the dialysis population than PIVA in that dialysis usually occurs in the outpatient setting where additional peripheral intravenous access is typically not present. Previously, we have demonstrated that NIVA signals could be collected with good correlation over a wide range of pulmonary capillary wedge pressures (r = 0.69, *n* = 83, PCWP = 4–40 mmHg), which is reflective of left ventricular filling pressures and considered by clinicians to be the gold-standard measure of volume status [16]. Additionally, NIVA has demonstrated sensitivity in the detection of 500 mL blood loss (AUC = 0.94) [15].

Here we demonstrate an observational study for a potential application of NIVA in assessing the volume status of patients undergoing hemodialysis. We hypothesized that there will be a significant change in pre-dialysis NIVA values when compared to post-dialysis NIVA values and that the intra-dialytic rate of change NIVA values will be predictive of an IDH episode.

## Methods

This study was performed in accordance with the Vanderbilt University Medical Center Institutional Review Board and due to potential institutional conflicts, the University of Alabama Institutional Review Board. Fifty patients admitted to the hospital for various conditions and also receiving intermittent HD (Fresenius 2008 Series Dialysis Machines, Waltham, MA, USA) were enrolled and informed consent obtained. The dialysate content, ultrafiltration rate, and goal for volume removal were determined by the nephrologist based on clinical assessment, per routine. Standard dialysate temperature was 37 °C.

Exclusion criteria from enrollment included pre-dialysis hypotension (MAP < 55 mmHg), mechanical ventilation, active atrial fibrillation, infiltrative cardiomyopathies, severe cardiac valve disease, congenital heart disease, decompensated moderate-severe congestive heart failure, or cardiac mechanical assist devices. After enrollment, a 10-mm piezoelectric sensor was secured to the volar aspect of the wrist on the upper extremity opposite to the dialysis access site with Coban Self-Adherent Wrap (3 M, US). All patients were in the semi-recumbent position and instructed to keep the upper extremity still. Venous signals were continuously acquired fifteen minutes before, throughout, and up to fifteen minutes after hemodialysis. Venous signals were analyzed in the time periods prior to and post-dialysis, when the dialysis pump was paused. Venous signals were also analyzed throughout dialysis in patients when the signal to noise ratio (SNR) was > 20. Standard vital signs including pulse rate, non-invasive cuff arterial blood pressure, respiratory rate and oxygen saturation were measured using an IntelliVue bedside monitoring system (Phillips North America Corp, Andover, MA, USA). Blood pressure measurements were obtained every 15 min per standard HD protocol.

**Fig. 2:**
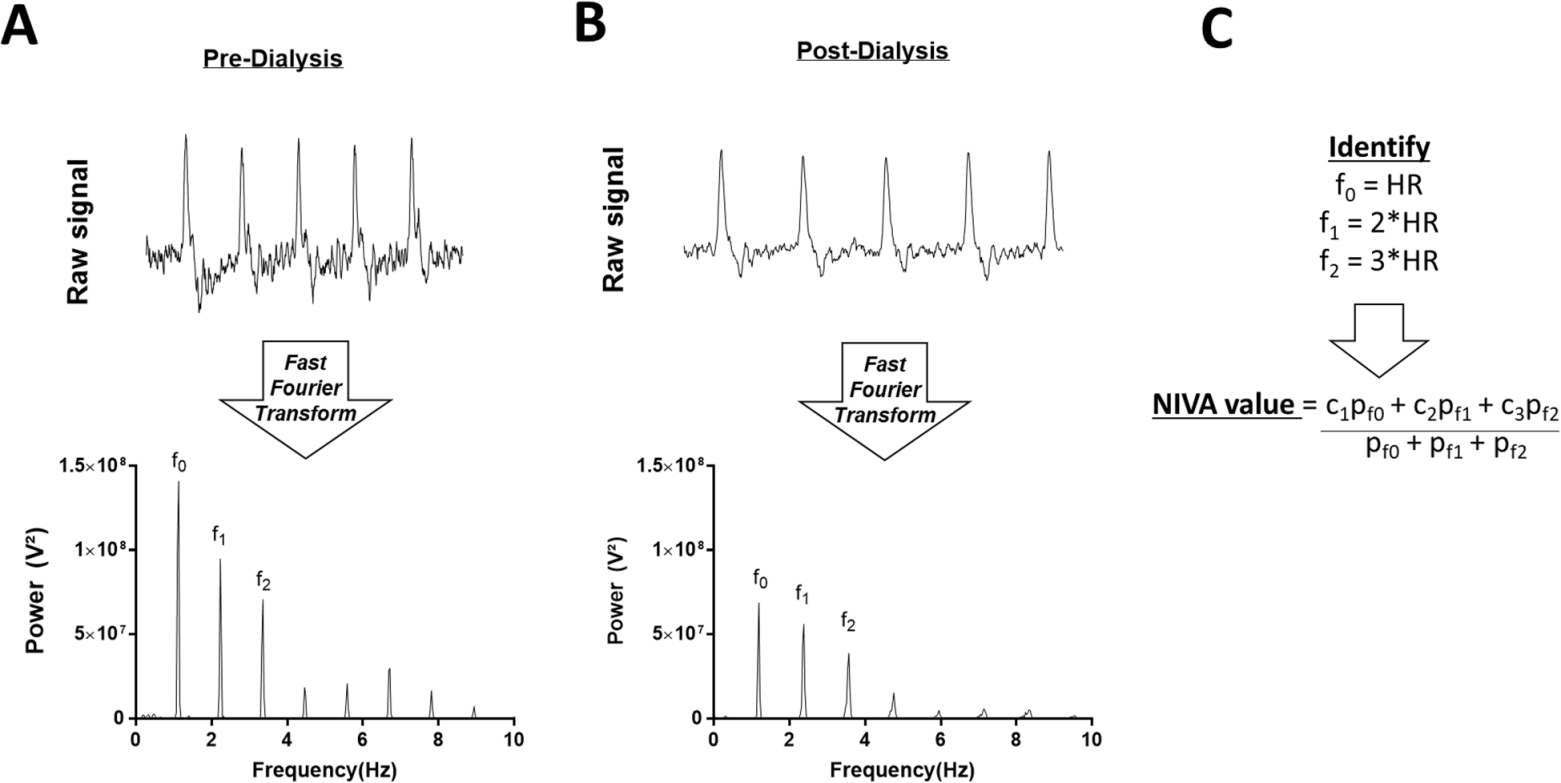
Representative waveforms in the time and frequency domains. Raw waveform (top) and fast Fourier transform (bottom) of signals taken **(a)** prior to and **(b)** post-dialysis. **(c)** Description of technique for calculation of a NIVA value. c_1–3_ are weighted constants, p_f0-f2_ represent the powers of f_0_-f_2_

Analysis of the venous signal was performed after a fast Fourier transformation (FFT) of the signal from the time to the frequency domain using 16 k windows utilizing an automated script written in C++. Data was recorded at a sampling rate of 500 Hz necessitating 32 s of continuous time-domain signal to perform the 16 K-FFT spectral analysis. The automated script locates the pulse rate from the signal and subsequent peaks in the spectral domain and is able to determine a signal to noise ratio to validate acceptable signals. A proprietary weighted algorithm was used to derive a NIVA value from the power spectrum of the frequency domain (Fig. 2c). Data points were generated by averaging three to five NIVA values derived from non-overlapping, low-noise segments of signal which achieved a signal to noise ratio greater than 20.

Statistical analysis was performed with JMP Pro 11 (Cary, NC, USA) and GraphPad Prism (La Jolla, Ca, USA). Two-tailed, paired t-tests were utilized to analyze significant changes in NIVA value pre- and post- dialysis.

In patients with signals of acceptable quality throughout dialysis, a least-squares model was fit for each individual subject to evaluate changes in normalized NIVA values over time. NIVA values were normalized by defining 0% as the first or last value, whichever was smaller, and defining 100% as the largest value in the set. In the subset of these patients with IDH, defined as a decrease in SBP > 20 mmHg or a decrease in MAP > 10 mmHg, a segmental least-squares model was also fit prior to and after the onset of IDH. The slopes between the two time groups were compared using a nonparametric Wilcoxon matched-pairs signed rank test. Overall slopes were compared in patients who experienced IDH versus those who did not using a nonparametric Mann-Whitney test for unpaired data. A Mann-Whitney test was also used to compare the overall slopes of patients who did not experience IDH to the segmental slopes prior to the onset of IDH in patients who experienced IDH. The ability of the latter to predict onset of IDH was evaluated with a receiver operating curve.

## Results

Fifty patients met inclusion criteria and were enrolled in the study. Seventy six percent of subjects were included in the analysis. Seven patients were excluded due to insufficient end-point data either related to early termination of dialysis or early removal of the device. Three patients were excluded due to a very low signal to noise ratio for the entirety of the waveform capture. Two patients were excluded from the final analysis as their change in NIVA values were considered outliers. Outliers were defined as values > 1.5 times the interquartile range below the first quartile or above the third quartile. Body mass index (BMI) did not significantly affect SNR (Supplemental Figure 1).

Demographic information for analyzed subjects is presented in Table 1**.** A higher proportion of males were analyzed (60%), but this was not statistically significant (*p* = 0.19). Mean and median age were 59.6 and 60 years respectively, with a range from 33 to 85 years. Net fluid removed followed a normal distribution with a mean of 2.27 l of net fluid removed. HD duration, on the other hand, followed a more asymmetrical distribution with a mean and median of 209 and 231 min, respectively.

**Table 1:** Demographic information

	^**1**^N (%) ^**2**^Mean (SD)	***P***-value
**Gender**^1^		
Male	23 (60%)	
Female	15 (40%)	
All	38 (100%)	
**Age**^2^ (yrs)	58.5 (12.9)	
**BMI**^2^ (kg/m^2^)	29.3 (6.9)	
**Comorbidities**^**1**^		
Heart Failure	24 (63%)	
Diabetes	20 (53%)	
Hypertension	31 (82%)	
**Heart rate (bpm)**^**2**^		
Pre-HD	79 (14)	*p* = 0.64
Post-HD	81 (13)	
**Mean arterial blood pressure (mmHg)**^**2**^		
Pre-HD	92 (17)	*p* = 0.32
Post-HD	88 (17)	
**Net fluid removed**^2^ (L)	2.27 (0.99)	
**HD duration**^2^ (min)	209.2 (44.5)	
**Pre-HD NIVA**^2^		
Male	1.20 (0.37)	*p* = 0.88
Female	1.21 (0.34)	
All	1.20 (0.35)	
**Post-HD NIVA**^2^		
Male	0.87 (0.31)	*p* = 0.95
Female	0.87 (0.22)	
All	0.87 (0.27)	

^1^N (%)

^2^Mean (SD)

**Fig. 3:**
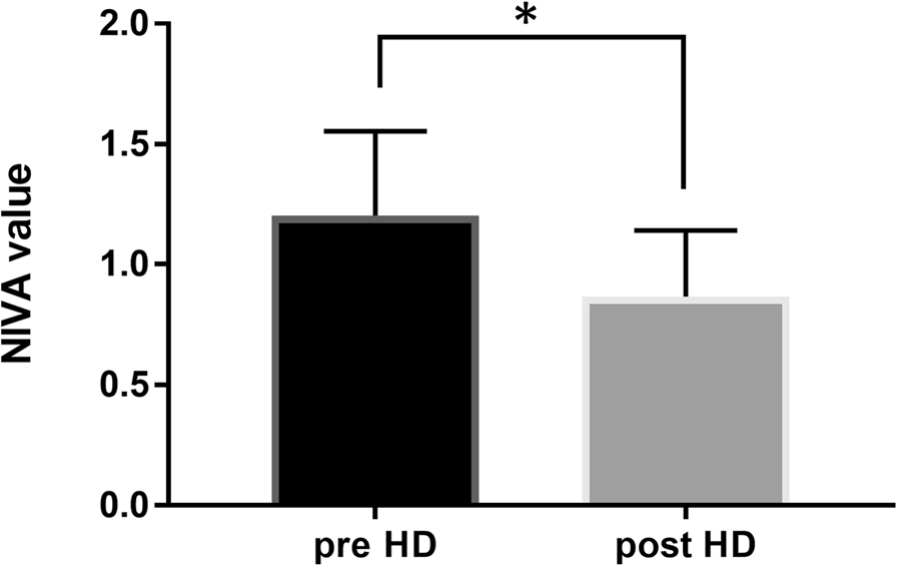
Average NIVA value pre- and post- HD. NIVA values decreased significantly after dialysis compared to pre-dialysis levels (*p < 0.05, *n* = 38)

NIVA values were significantly lower at the end of dialysis compared to pre-dialysis levels, with a mean of 1.203 prior to dialysis compared to a mean of 0.868 post-dialysis (*p* < 0.05, Fig. 3).

**Fig. 4:**
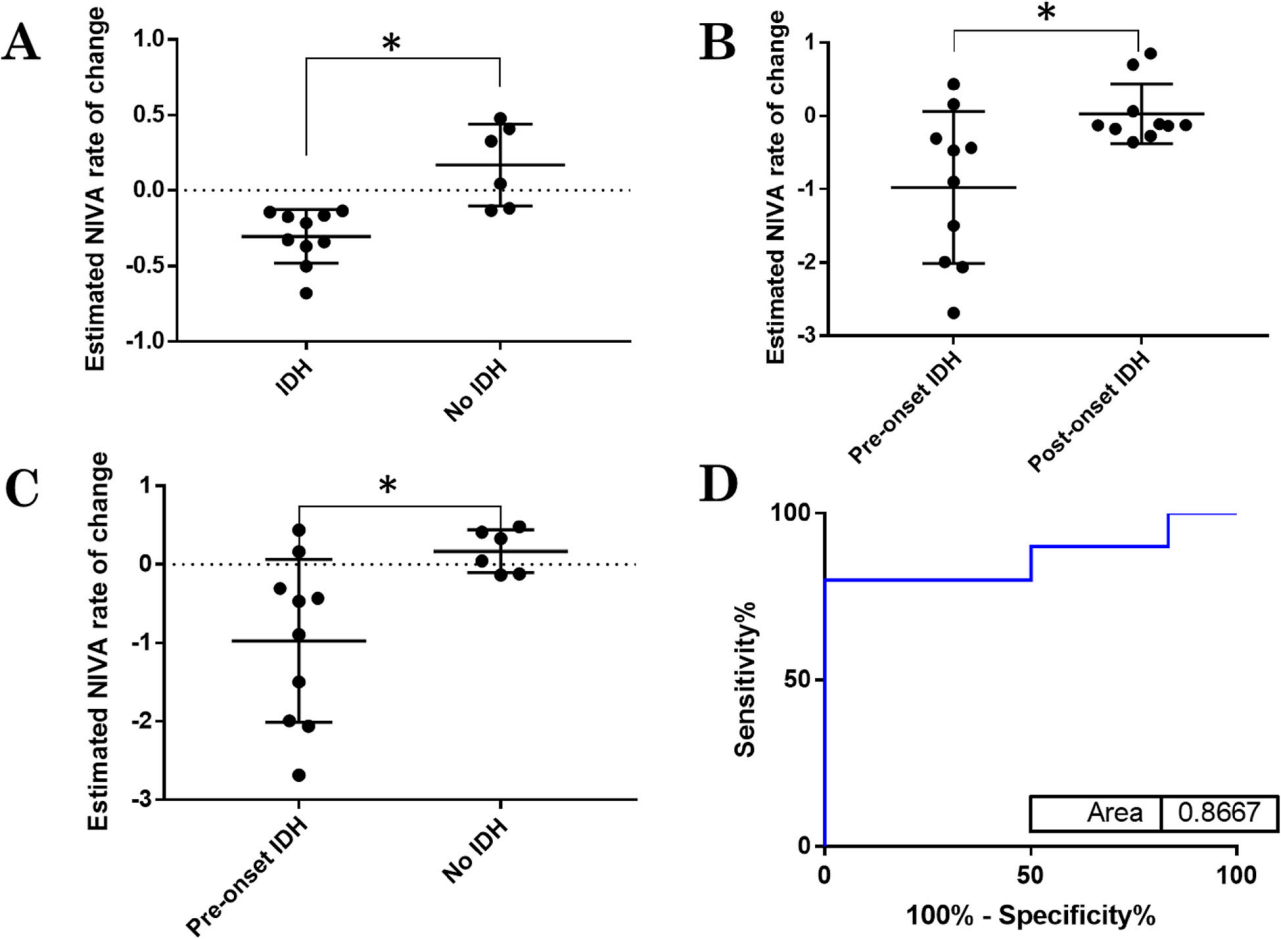
Prediction of IDH with changes in NIVA values and heart rate. (**a**) Slopes of the least-squares model for NIVA over time were significantly steeper in patients with IDH. (**b**) Slopes of the least-squares model for NIVA over time were significantly steeper prior to the onset of IDH in patients with IDH. IDH occurred at a mean dialysis time of 68 min (range 15–210 min) (**c**) Slopes of the least-squares model for NIVA over time were significantly steeper prior to the onset of IDH in patients with IDH compared to patients without IDH (D) These slopes were able to predict the *onset* of IDH with an AUC = 0.87, sensitivity of 80% and specificity of 100% (*n* = 16)

Of the 38 patients analyzed, 16 patients had data obtained throughout their dialysis session that was of sufficient quality for analysis, based on a signal to noise ratio > 20. Of these 16 patients, 10 (62.5%) met the predefined criteria for IDH at some point during their dialysis session. The change in NIVA value over the entire dialysis session was estimated by fitting a least squares model to the graph of normalized NIVA values versus time and comparing the slopes (β) of these models. The slopes of these lines were significantly steeper in those patients who had an episode of IDH versus those who did not (*p* < 0.05**,** Fig. 4a). In the patients who experienced IDH, when a segmental least-squares model was fit in the time periods prior to the onset of IDH and after the onset of IDH, the slopes were significantly steeper prior to the onset of IDH (*p* < 0.05, Fig. 4b). The segmental slope prior to the onset of IDH was steeper compared to the overall slopes of patients without IDH (*p* < 0.05, Fig. 4c), with a slope of < − 0.22 having 80% sensitivity and 100% specificity to predict the onset of IDH (AUC 0.87, *p* < 0.05, Fig. 4d).

## Discussion

Hemodynamic changes such as intradialytic hypotension result from rapid ultrafiltration of excess volume during hemodialysis and have important implications on morbidity and mortality in the hemodialysis-dependent population. Overall 5-year survival in HD patients is approximately 40%, with cardiovascular disease including heart failure, cardiac ischemia, and arrhythmias responsible for almost 50% of deaths among dialysis patients [1]. HD results in a pronounced reduction in myocardial perfusion, related both to decreased cardiac output and failed sympathetic compensatory mechanisms in both symptomatic and asymptomatic patients [23, 24]. Intradialytic hypotension (IDH) due to compromise of cardiovascular hemodynamics during fluid removal is the most common serious complication of dialysis, occurring in up to 30% of HD sessions, and further potentiates cardiac, splanchnic, and cerebral end-organ hypoperfusion [3, 5, 6]. Furthermore, IDH often results in premature termination of HD sessions resulting in inadequate volume and solute removal. These negative consequences of IDH for the patient persist despite the use of clinical parameters to estimate volume status and adequate ultrafiltration requirements, such as current versus dry weight, peripheral edema, jugular venous distention, vital signs including mean arterial pressure and heart rate, lung auscultation and subjective shortness of breath [3].

A clear need exists for appropriate technology to better assist clinicians with volume status assessment both prior to and during dialysis to balance maintenance of adequate cardiac output and end-organ perfusion with appropriately maximized volume removal to reach a state of euvolemia. To date, proposed techniques to guide volume-directed dialysis have included continuous blood volume measurements, bioelectrical impedence measurements, thoracic admittance measurements, and analysis of photoplethysmograph signals [25]. However, these monitoring techniques have not been successfully implemented into widespread clinical practice and most do not show consistent correlations with IDH. Here, we describe a novel method, NIVA, to noninvasively obtain and analyze a previously overlooked physiologic signal - the peripheral venous waveform.

This proof-of-concept observational study utilizing an early prototype suggests that, under certain conditions, it is possible for NIVA to obtain peripheral venous waveforms during dialysis. The significant decrease in NIVA values after dialysis suggests that NIVA may successfully identify changes in fluid status after dialysis. With improved signal acquisition, future applications of the NIVA device may better define target NIVA values representative of euvolemia in patients with ESRD. Although such a value would need to be individualized and determined in conjunction with other clinical findings, preliminary data supports the premise that NIVA values representative of euvolemia are lower than NIVA values recorded pre-dialysis (Supplemental Figure 2).

Additionally, in a small subset of patients, signals were successfully able to be obtained during dialysis allowing for analysis of the dynamic changes in NIVA value related to IDH. In this admittedly small cohort, a faster drop in NIVA values occurred in patients with IDH. Within patients with IDH, least-squares slopes of NIVA values were steeper prior to the onset of IDH. After the onset of IDH, patients are often given fluid boluses and/or ultrafiltration rates are lowered, which likely explains the change in slope magnitude noted after the onset of IDH. Comparison of the slope prior to the onset of IDH to the overall slopes of patients without IDH had high sensitivity toward the prediction of onset of IDH (80% sensitivity and 100% specificity, AUC 0.87, *p* < 0.05).

There are a number of limitations in this observational study. First, our subjects were limited to the acutely ill inpatient population. A much larger study will need to be performed to help better understand the relationship of various comorbidities with the NIVA value. There were not enough patients to perform a subgroup analysis to identify specific patient populations/pathologies that may preclude NIVA. Additional treatment data, including dry weights and frequency of dialysis treatments may also be useful to examine in future studies to gain a better understanding of the subject’s overall starting volume status. Blood and dialysate flow rates may be useful to analyze in relation to NIVA signal noise. Second, there was a lack of information regarding previous dialysis access or venous duplex studies. Patients with ESRD have often had multiple access operations in both upper extremities, and the effects of failed access, venous stenosis, or venous thrombosis on the distal peripheral venous waveform is unknown. Similarly, venous calcification, though less common than arterial calcification in patients with ESRD, is possible and the effect on NIVA signal acquisition is currently unknown [26, 27]. Third, the effects of body temperature on NIVA signals is not currently known. Although the fundamental origin of the signal obtained with the NIVA device, which is placed directly over the superficial veins of the wrist, is venous, the signal is obtained at the surface of the skin and the effects of varying mechanical properties of the interposing tissue from edema is unknown and currently subject to ongoing study. Of note, the amount of tissue between the superficial veins, which are often visible, is small, and BMI did not significantly affect SNR (Supplemental Figure 1). Lastly, although the results regarding the ability of dynamic NIVA changes to predict IDH are promising, they are highly limited by the small sample size. 12 out of 50 (24%) of enrolled patients were excluded for various reasons, and only 16 out of 50 patients had acceptable quality signal during dialysis so there is a possibility of selection bias in analysis of the data and larger studies are needed. One-third of excluded subjects were secondary to insufficient battery life, representing an area for potential improvement with future device iterations. Additionally, the venous waveform is a low amplitude signal, and signal quality is sensitive to noise from movement and potentially mechanical noise originating from the dialysis machine. There is likely also some variability in the counter-pressure of the sensor on the skin with application, which may affect signal acquisition. One excluded subject requested the device be removed early secondary to discomfort. Sensors were applied with the intent to be secure enough to detect the venous pulsations with minimal noise, while preventing venous compression. Future testing will allow determination of the optimal pressure of application for signal acquisition and comfort, with a goal of incorporating a dedicated wristband to the sensor and a mechanism to detect when adequate pressure has been achieved. Further development toward signal amplification to decrease the effect of noise is currently underway, and future iterations of the device will be able to capture a larger sample size of subjects with useable NIVA signal throughout their dialysis session. This will allow future studies to better understand the dynamic changes in NIVA signal during dialysis and how they may correlate to IDH or intradialytic symptoms such as muscle cramping. Finally, NIVA signals have not been accurately obtained in patients with atrial fibrillation, infiltrative cardiomyopathies, severe cardiac valve disease, congenital heart disease, or with cardiac mechanical assist devices. These subsets of patients also include those with ESRD and a better understanding of how each of these unique cardiac states affect the NIVA signal is needed. Although signals have been successfully obtained in patients with decompensated heart failure and undergoing mechanical ventilation, for this study these populations were excluded from enrollment to limit potential confounding effects on the venous signal.

## Conclusions

In conclusion, in this proof-of-concept observational study, NIVA signals were successfully obtained before, during, and after HD using an early prototype device. Further development, with particular emphasis on improvement of signal quality, may provide static and continuous measures of volume status on a larger proportion of patients undergoing dialysis. This will allow for a more robust analysis on the predictive value of NIVA in regards to IDH; future studies will also aim to examine the correlation between change in NIVA value with ultrafiltration volumes. This information may ultimately be developed to guide amount and rate of ultrafiltration volume removal during dialysis by: (1) identifying an accurate and attainable baseline or target euvolemic state; (2) accurately characterizing volume status pre-dialysis; and (3) identifying excessive changes in volume removal during HD before hypotension and end-organ compromise occur. Taken together, NIVA is a novel non-invasive device with potential to support safe and effective volume directed dialysis.

## Supplementary information

**Additional file 1: Supplemental Figure 1.** Signal-to-noise ratio (SNR) averaged over signal acquisition time plotted as a function of body mass index (BMI). There is no significant correlation between BMI and SNR (r = 0.01, *p* = 0.93). **Supplemental Figure 2.** NIVA values were obtained on 10 healthy control subjects (5 males, 5 females) under institutional IRB approval. Mean age was 30 years with a range of 19–58 years. Signal acquisition and analysis was performed in an identical manner as described in the methods. A Mann-Whitney test was used to compare NIVA values of control subjects to those of subjects pre-dialysis (*n* = 38). NIVA values of euvolemic controls were significantly lower than NIVA values pre-dialysis (median 0.99 vs 1.22, *p* = 0.03).

## Data Availability

The data that support the findings of this study are available from Volumetrix but restrictions apply to the availability of these data, which were used under license for the current study, and so are not publicly available. Data are however available from the authors upon reasonable request and with permission of Volumetrix.

## Abbreviations

BMI: Body mass index
ESRD: End stage renal disease
FFT: Fast Fourier transform
HD: Hemodialysis
IDH: Intradialytic hypotension
KDOQI: Kidney Disease Outcomes Quality Initiative
NIVA: Non-invasive venous waveform analysis
PCWP: Pulmonary capillary wedge pressure
PIVA: Peripheral intravenous waveform analysis
SNR: Signal-to-noise ratio
