# Supplementary material for: Non-invasive venous waveform analysis (NIVA) for volume assessment in patients undergoing hemodialysis: an observational study
Source: BMC Nephrol. 2020 May 24;21:194. doi: 10.1186/s12882-020-01845-2 (PMC7245891; doi:10.1186/s12882-020-01845-2)
Supplement: Supplementary file 1 — Additional file 1: Supplemental Figure 1. Signal-to-noise ratio (SNR) averaged over signal acquisition time plotted as a function of body mass index (BMI). There is no significant correlation between BMI and SNR (r = 0.01, p = 0.93). Supplemental Figure 2. NIVA values were obtained on 10 healthy control subjects (5 males, 5 females) under institutional IRB approval. Mean age was 30 years with a range of 19–58 years. Signal acquisition and analysis was performed in an identical manner as described in the methods. A Mann-Whitney test was used to compare NIVA values of control subjects to those of subjects pre-dialysis (n = 38). NIVA values of euvolemic controls were significantly lower than NIVA values pre-dialysis (median 0.99 vs 1.22, p = 0.03). [file 12882_2020_1845_MOESM1_ESM.docx]

**Supplemental Figure 1.** Signal-to-noise ratio (SNR) averaged over signal acquisition time plotted as a function of body mass index (BMI). There is no significant correlation between BMI and SNR (r=0.01, p=0.93)


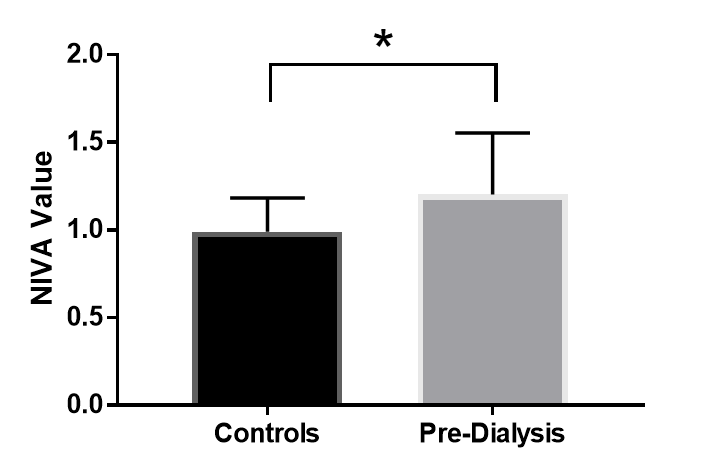


**Supplemental Figure 2.** NIVA values were obtained on 10 healthy control subjects (5 males, 5 females) under institutional IRB approval. Mean age was 30 years with a range of 19-58 years. Signal acquisition and analysis was performed in an identical manner as described in the methods. A Mann-Whitney test was used to compare NIVA values of control subjects to those of subjects pre-dialysis (n=38). NIVA values of euvolemic controls were significantly lower than NIVA values pre-dialysis (median 0.99 vs 1.22, p=0.03).
